# A Symbolic Inference System for AI Decision Support in Multimodal Assessment of Pediatric Cerebral Visual Impairment

**DOI:** 10.3390/s26216673

**Published:** 2026-10-22

**Authors:** Aleksandar Miladinović, Alessandro Biscontin, Miloš Ajčević, Agostino Accardo, Stefano Pensiero, Paola Michieletto

**Affiliations:** 1 Institute for Maternal and Child Health–IRCCS Burlo Garofolo, 34137 Trieste, Italy; alessandro.biscontin@burlo.trieste.it (A.B.); paola.michieletto@burlo.trieste.it (P.M.); 2 Department of Engineering and Architecture, University of Trieste, 34127 Trieste, Italy; majcevic@units.it (M.A.); accardo@units.it (A.A.); 3 Scientific Institute IRCCS Eugenio Medea, 31053 Pieve di Soligo, Italy; s.pensiero@libero.it

**Keywords:** cerebral visual impairment, symbolic artificial intelligence, clinical decision support, neurosymbolic reasoning, provenance, explainable artificial intelligence, multimodal assessment, pediatric ophthalmology

## Abstract

Cerebral visual impairment (CVI) is a heterogeneous neurodevelopmental condition in which visual dysfunction may affect acuity, visual field, ocular motility, fixation, electrophysiological responses, and functional vision. Multimodal assessment is therefore required, but the resulting evidence is often incomplete, heterogeneous, and difficult to integrate reproducibly. This computational feasibility study developed a symbolic AI decision-support model for multimodal assessment of pediatric CVI. Sixty-two children older than 18 months and younger than 18 years were encoded as patient-level facts after neuro-ophthalmological assessment and brain and orbital MRI. Structured variables included MRI status, refraction, ocular motility, visual field testing, and visual evoked potentials. An anterior-pathway-not-explanatory predicate was used as a non-weighted eligibility gate, while a priori expert-defined support weights were assigned to positive evidence domains. MRI abnormalities and ocular motility abnormalities were each present in 47/62 patients (75.8%), visual field alterations in 16/62 (25.8%), and altered visual evoked potentials in 22/62 (35.5%). At least one predefined CVI-compatible functional proof path was established in 54/62 patients (87.1%). With k=1, CVI support scores ranged from 0.700 to 0.800, reflecting the strongest retained functional path. With k=3, the maximum support score was 0.985 in patients with three retained functional paths. Equal-weight ablation (w=0.75 for each functional domain) preserved logical derivability and yielded a three-path value of 0.9844, indicating that multi-path convergence was not dependent on the small differences among the expert-assigned functional weights. No independent diagnostic gold standard was available; therefore, these findings characterize symbolic derivability and provenance behavior rather than diagnostic accuracy. The proposed system provides an explicit, provenance-aware decision-support representation and a foundation for future neurosymbolic integration of quantitative clinical and instrumental data.

## 1. Introduction

Cerebral visual impairment (CVI) is a spectrum of visual impairments caused by abnormal development or injury of visual processing pathways in the brain. The most recent working definitions emphasize deficits of visual function and functional vision that are not sufficiently explained by comorbid ocular disease alone [1,2]. CVI is now recognized as one of the major causes of childhood visual impairment in high-income countries and is frequently encountered in children with prematurity, perinatal brain injury, epilepsy, genetic syndromes, malformations, cerebral palsy, and other complex neurodevelopmental conditions [3,4,5].

A central difficulty in CVI assessment is phenotypic heterogeneity. Children may present with reduced visual acuity, visual field loss, impaired fixation, strabismus, nystagmus, abnormal ocular motility, crowding, visual search impairment, visuospatial dysfunction, or higher-order visual perceptual difficulties [3,6,7]. These findings do not define a single uniform phenotype. Clinical and research approaches therefore often separate visual acuity-related impairment, visual-field-related impairment, oculomotor or fixation impairment, electrophysiological pathway involvement, and higher-order dorsal or ventral stream dysfunction [7,8,9,10]. This distinction matters because relevant visual dysfunction may be present even when visual acuity is relatively preserved [7].

Current assessment relies on the integration of multiple information sources. Ocular examination and OCT can support the exclusion or characterization of retinal and optic nerve disease; refraction is required before interpreting acuity; visual field testing may reveal retrochiasmatic dysfunction; ocular motility assessment captures fixation and gaze abnormalities; electrophysiology provides objective information on visual pathway function; and MRI documents structural abnormalities affecting visual pathways or association networks [2,7,10,11]. Intervention and assessment reviews also emphasize the importance of documenting functional vision, neurological context, and the child’s real-world visual behavior [10,12]. In daily practice, however, these data are rarely complete. Test availability depends on age, cooperation, cognitive and motor abilities, and clinical indication.

This creates a practical decision-support problem. Clinicians must reason over heterogeneous evidence, distinguish anterior pathway disease from brain-based visual dysfunction, recognize converging evidence across domains, and identify discordant patterns that need review. A clinical decision-support system is intended to assist this process by matching patient-specific information with structured knowledge and presenting patient-specific assessments or recommendations to clinicians [13,14]. For pediatric CVI, the main requirement is not only prediction but traceability: clinicians need to know which findings support an output, which domains are missing, and whether the evidence comes from one dominant path or several converging paths [15,16].

Recent advances in artificial intelligence have shown the potential of neural networks and deep learning for extracting quantitative information from medical images and biomedical signals [17,18,19,20]. In ophthalmology and neuro-ophthalmology, such methods may be applied to OCT, corneal topography, ocular ultrasound, MRI, eye-tracking recordings, electroretinography, electrooculography, and visual evoked potentials. Nevertheless, deep-learning outputs are difficult to use clinically if they remain isolated from transparent reasoning structures. This is why several authors have emphasized human-centered validation, interpretability, and workflow integration for medical AI and decision support [21,22,23,24].

To move toward a multimodal AI approach for pediatric CVI, a symbolic knowledge and inference layer is needed. Such a layer can represent current clinical reasoning, encode structured findings as patient-level facts, assign expert-defined support weights, and expose the proof paths that lead to a given output. Symbolic inference is suitable for this role because it allows explicit clinical rules, weighted evidence, and provenance-based interpretation [13,14,25,26]. In this framework, the system is transparent because the rules are explicitly defined, interpretable because each output is linked to clinically meaningful evidence domains, and provenance-aware because the supporting proof paths can be inspected.

From a sensing perspective, the proposed framework can be regarded as a symbolic fusion layer positioned downstream of heterogeneous ophthalmic, neuroimaging, eye-movement, and electrophysiological instruments. In the present retrospective implementation, examination results were represented as structured patient-level facts rather than being extracted directly from raw sensor streams. The same architecture can nevertheless receive continuous or probabilistic features generated from video-oculographic eye-position recordings, OCT images, MRI-derived measurements, and electrophysiological waveforms. In this way, the symbolic layer can connect instrument-derived measurements with clinically defined reasoning paths while retaining the provenance of each output.

A related example from ophthalmic AI is our previous work on OCT-based retinal disease classification, where image-derived evidence was combined with expert-defined symbolic reasoning to improve interpretability [27]. This line of work has recently been extended by Inferrera et al. to spatially grounded neurosymbolic OCT classification, integrating retinal-layer segmentation with neural sign extraction and symbolic clinical reasoning [28]. The present study is independent from these OCT studies and addresses a different clinical problem: the use of a symbolic inference layer to organize heterogeneous clinical and instrumental evidence in pediatric CVI assessment.

Therefore, the aim of this study was to develop and test a symbolic AI inference system for decision support in the multimodal assessment of pediatric CVI. The system encodes patient findings as weighted facts, applies expert-defined symbolic rules, and returns CVI-compatible support profiles with top-k provenance. The output is intended to organize multimodal evidence, expose supporting proof paths, and assist clinical review. It is not intended to provide an autonomous diagnosis or calibrated probability of CVI.

## 2. Materials and Methods

### 2.1. Study Population

This retrospective study included a cohort of 62 pediatric patients: 39 males and 23 females, with a mean age of 6.4 ± 4.2 years. The cohort included children with complex neurological or neurodevelopmental conditions who underwent neuro-ophthalmological assessment and brain and orbital MRI. For the present analysis, the available ocular and neuro-ophthalmological findings were reviewed to determine whether ocular, retinal, optic nerve, or chiasmal abnormalities were sufficient to explain the documented visual dysfunction. This determination was represented within the inference model as an eligibility gate rather than as positive evidence for CVI. MRI abnormalities were present in 47 patients, whereas 15 patients had no MRI abnormalities. The main clinical and etiological characteristics of the cohort are summarized in Table 1.

Written informed consent was obtained from a parent or legal guardian before participation. The study protocol was approved by the Institutional Scientific Board of IRCCS Burlo Garofolo (RC 31/24) and was conducted in accordance with the Declaration of Helsinki.

### 2.2. Reference Standard and Intended Evaluation

No single instrumental gold standard is currently available for the diagnosis of pediatric CVI. Diagnosis is based on clinical synthesis of visual dysfunction, neurological history, ocular and neuro-ophthalmological findings, functional vision, and complementary instrumental examinations [2,5,7,10]. The present study was therefore designed as a computational feasibility study rather than as a diagnostic-accuracy study.

The symbolic output was not compared with an independent binary reference diagnosis. The primary evaluation concerned whether the encoded patient findings established predefined CVI-compatible evidence paths and how multiple retained proof paths affected provenance-based support values. Consequently, the proportion of records with a derived path should be interpreted as a descriptive derivability rate within this cohort and not as sensitivity, specificity, diagnostic accuracy, estimated disease prevalence, or demonstrated clinical utility. A statistical or machine-learning diagnostic baseline was not fitted because no independent diagnostic target was available; training a comparator against the rule-derived output would constitute circular validation rather than an independent performance comparison. The binary 54/62 summary can also be reproduced by directly counting records with at least one qualifying functional finding and is therefore used only as a descriptive cohort summary. The added computational information evaluated here lies beyond this binary endpoint: the symbolic program represents intermediate predicates and alternative derivations, while provenance records which proof paths support an individual inference. A checklist/direct-count comparison and targeted provenance ablations are reported in Supplementary Section S4 (Table S1).

The frozen symbolic implementation was subjected to a final reproducibility check using the Scallop command-line interface scli version 0.1.4. The rerun reproduced 54/62 CVI-compatible functional relations and the patient-level top-*k* outputs reported below; the corresponding mean support values were 0.791, 0.858, and 0.862 for k=1, k=2, and k=3, respectively. The higher-level relations were also reproduced, including 25 records with at least two functional domains, 19 with MRI plus at least two functional domains, 5 with MRI plus all three functional domains, 12 MRI/function discordance profiles, and 22 VEP/function discordance profiles. Executable rules, commands, aggregate checks, and archived raw Scallop outputs are provided in Supplementary Sections S11 and S12; layered and contextual relation counts are summarized in Supplementary Section S9 (Table S4).

### 2.3. Clinical and Instrumental Variables

The following domains were considered for structured data extraction: demographic data, etiological category, structural MRI status, OCT imaging, refractive error, visual acuity, ocular motility, strabismus, nystagmus or fixation abnormalities, visual field status, and visual evoked potentials. Table 2 summarizes their structured representation.

Visual acuity was assessed using the method most appropriate for age and collaboration. Numerical values were expressed in decimal notation when obtained monocularly using optotype charts or comparable procedures. Refractive errors were coded as astigmatism, hyperopia, or myopia according to predefined severity thresholds.

Ocular motility abnormalities included strabismus, nystagmus, and evident fixation abnormalities. Strabismus was classified as exotropia, esotropia, or vertical strabismus when present. Visual field status was derived from the available clinical field examination, mainly Goldmann perimetry and attraction visual field testing (MonCvONE-PRO full-field projection perimeter; Metrovision, Pérenchies, France). Automated Humphrey perimetry was recorded when available but was not used as a separate rule-domain because it was feasible only in a small subset of patients. Visual evoked potentials included flash VEP and pattern VEP when available.

MRI findings were retrieved from the patients’ electronic health records and were based on the original clinical radiological reports. For the present study, Stefano Pensiero and Paola Michieletto reviewed the reported abnormalities in relation to the visual pathways and the overall CVI assessment. The MRI variable used in the rule base therefore represented the presence or absence of a clinically relevant structural abnormality rather than the presence of any incidental neuroradiological finding.

OCT images were collected when available using the RTVue XR Avanti System (Optovue, Inc., Fremont, CA, USA). OCT was considered as structural ocular and retinal imaging, used to document retinal and optic nerve status and to support exclusion or characterization of ocular contributions to visual impairment. Cycloplegic refraction was routinely used in the pediatric examinations. Assessment of whether ocular or anterior visual pathway abnormalities could sufficiently explain the visual dysfunction considered refractive findings, the general retinal examination, and OCT imaging where available, together with the available electrophysiological and neuroimaging information.

VEP examinations were acquired by trained technicians within the Ophthalmology Department using a Retimax clinical system (CSO Italia, Scandicci, Italy). The CE-marked device provides reference indicators based on patient information and measured electrophysiological parameters, including latency, to support interpretation. Flash and pattern VEP findings were subsequently reviewed in their clinical context by Pensiero and Michieletto. Because the study was retrospective, no additional numerical VEP threshold was imposed retrospectively beyond the clinical classification available from the examination. Pensiero and Michieletto reviewed the multimodal findings as part of ordinary clinical assessment and were not blinded to the other available clinical information; these findings are therefore treated as components of the multimodal representation rather than independent blinded reference standards.

### 2.4. Rule and Weight Construction

The symbolic rules were defined a priori, independently of analysis of the present cohort, by Stefano Pensiero and Paola Michieletto on the basis of their clinical expertise and the published clinical literature and recommendations for CVI assessment. The clinical concepts were subsequently reduced to explicit predicates and implemented in Scallop by Aleksandar Miladinovic and Alessandro Biscontin; the resulting implementation was then jointly reviewed with Pensiero and Michieletto to verify correspondence between the computational predicates and the intended clinical reasoning. No published CVI guideline specifies numerical weights for these evidence domains. The support weights were therefore assigned a priori by Pensiero according to their relative clinical relevance and were fixed before analysis of the present cohort. They are design parameters of the decision-support model rather than empirically calibrated disease probabilities. Robustness to these choices was examined using both local ±10% perturbation and an equal-weight ablation in which all three functional weights were set to 0.75 (Supplementary Section S8). The rule and weight construction received internal clinical review by the study clinicians, but no independent external expert validation was performed.

### 2.5. Missing, Normal, and Discordant Evidence

A recorded abnormal finding generated the corresponding positive evidence fact. A documented normal finding was represented separately when required by a discordance rule. An examination with no recorded result did not generate either a positive or a negative fact and therefore contributed no evidence to a rule requiring that domain. Because the retrospective source table does not consistently distinguish why an entry is absent (e.g., examination not performed, unavailable, or unreliable), these reasons were not inferred retrospectively. Examination availability is reported descriptively in Supplementary Table S3.

The anterior_pathway_not_explanatory(P) predicate was an eligibility condition rather than positive evidence. It represented the clinical judgement, based on review by Pensiero and Michieletto of the available ophthalmological and instrumental findings, that ocular or anterior visual pathway abnormalities were insufficient to explain the observed visual dysfunction. All 62 records in the present selected cohort satisfied this predicate. Logically, if the predicate is false, rules requiring it cannot be derived regardless of downstream functional evidence. A formal description of this behavior and examples involving missing, normal, and discordant evidence are provided in Supplementary Sections S2, S3 and S7.

### 2.6. Sensor and Signal Integration Framework

**Figure 1:**
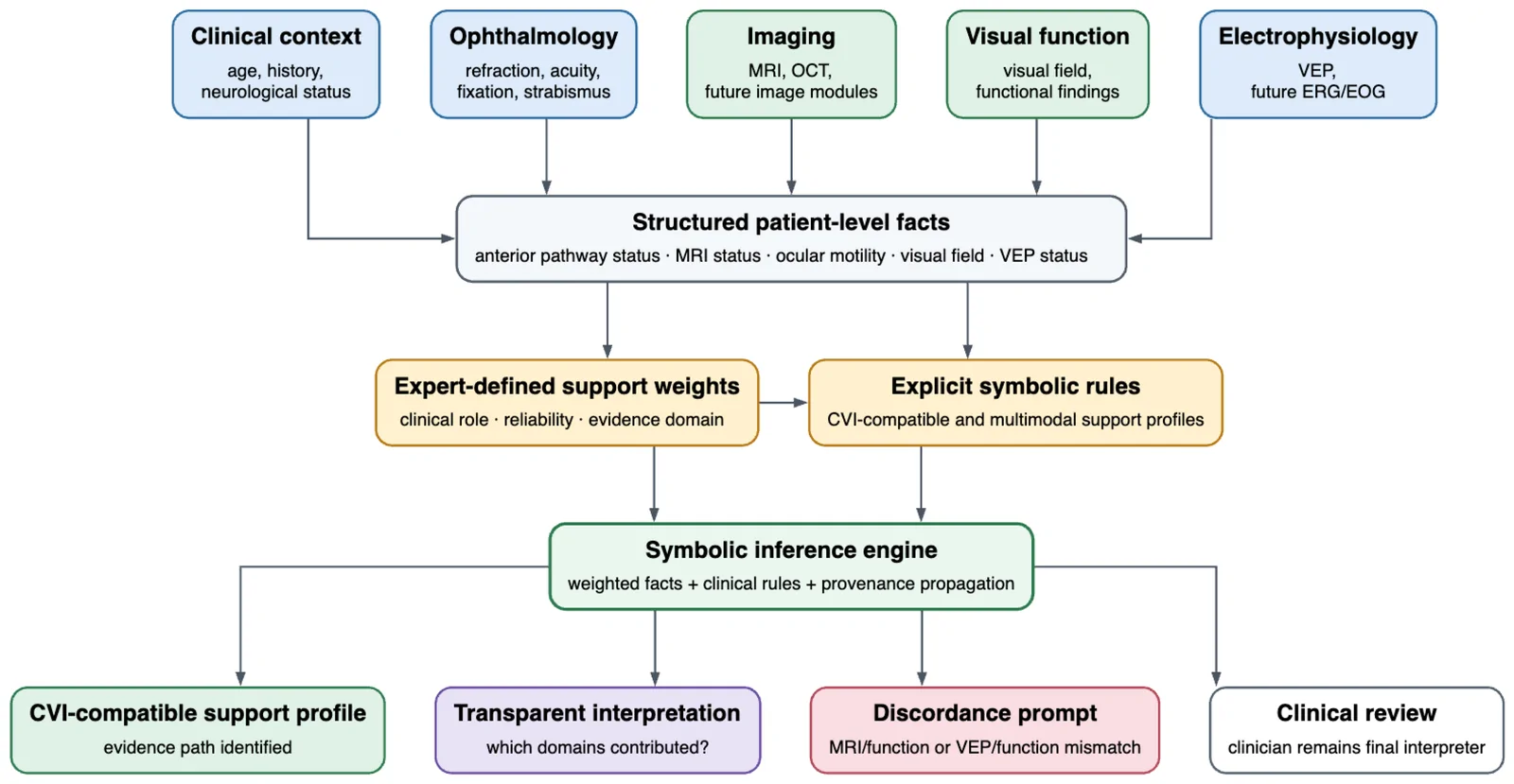
Workflow of the symbolic inference system for AI decision support in pediatric cerebral visual impairment. Multimodal clinical, ophthalmological, imaging, visual function, and electrophysiological data are encoded as structured patient-level facts. Expert-defined support weights and explicit symbolic rules are processed by the symbolic inference engine using top-k proof provenance. The system generates CVI-compatible support profiles, transparent interpretations, discordance prompts, and outputs intended for clinical review rather than autonomous diagnosis.

The proposed architecture was designed as a modular inference layer for heterogeneous clinical and instrumental information. In the present retrospective implementation, the inference rules operated on structured findings extracted from clinical records and instrumental reports. Direct rule inputs were MRI status, ocular-motility/fixation abnormality, visual-field alteration, VEP alteration, and the anterior-pathway-not-explanatory eligibility predicate. OCT, refraction, visual acuity, and other recorded examinations contributed to clinical characterization and assessment of anterior-pathway explanations but did not directly contribute numerical support to the reported top-*k* scores. Direct extraction of continuous or probabilistic facts from raw sensor streams was not evaluated in the present study and is considered a future extension. Figure 1 summarizes the inference workflow.

Three sensor pathways are particularly relevant for pediatric CVI assessment. First, high-resolution video-oculography can provide quantitative measurements of fixation stability, saccades, smooth pursuit, and ocular-following responses. Ocular-following responses are short-latency reflexive eye movements evoked by large-field visual motion. Motion stimuli, including unikinetic plaid patterns, may be used to probe the integration of one-dimensional and two-dimensional motion signals and to derive quantitative features associated with cortical motion processing. Such measurements could be encoded together with recording quality and confidence estimates.

Second, raw OCT B-scans and volumetric acquisitions could be processed using image-analysis or deep-learning methods to identify retinal and optic nerve features. Potential inputs include retinal nerve fiber layer thickness, ganglion cell complex thickness, segmentation abnormalities, image-quality indices, and structural patterns that may indicate retinal disease or trans-synaptic degeneration. Rather than relying only on a binary OCT interpretation, these outputs could be represented as weighted facts linked to their source images and algorithmic confidence.

Third, electrophysiological recordings could be incorporated at the waveform level. VEP latency, amplitude, interocular asymmetry, reproducibility, and signal-quality indices could be represented as continuous or probabilistic inputs. The same approach could subsequently include electroretinography and electrooculography when clinically available. In this configuration, the symbolic inference engine would combine sensor-derived features with clinical context while retaining the proof path linking each output to its originating measurement. Table 3 summarizes potential sensor-derived features and their roles.

### 2.7. Symbolic Inference Architecture

The reasoning module was formulated as a declarative probabilistic logic program implemented through a symbolic inference engine. Each patient-level observation was encoded as a structured fact, with the patient identifier denoted by *P*. Examples included MRI status, visual field alteration, ocular motility abnormality, and VEP alteration.

**Figure 2:**
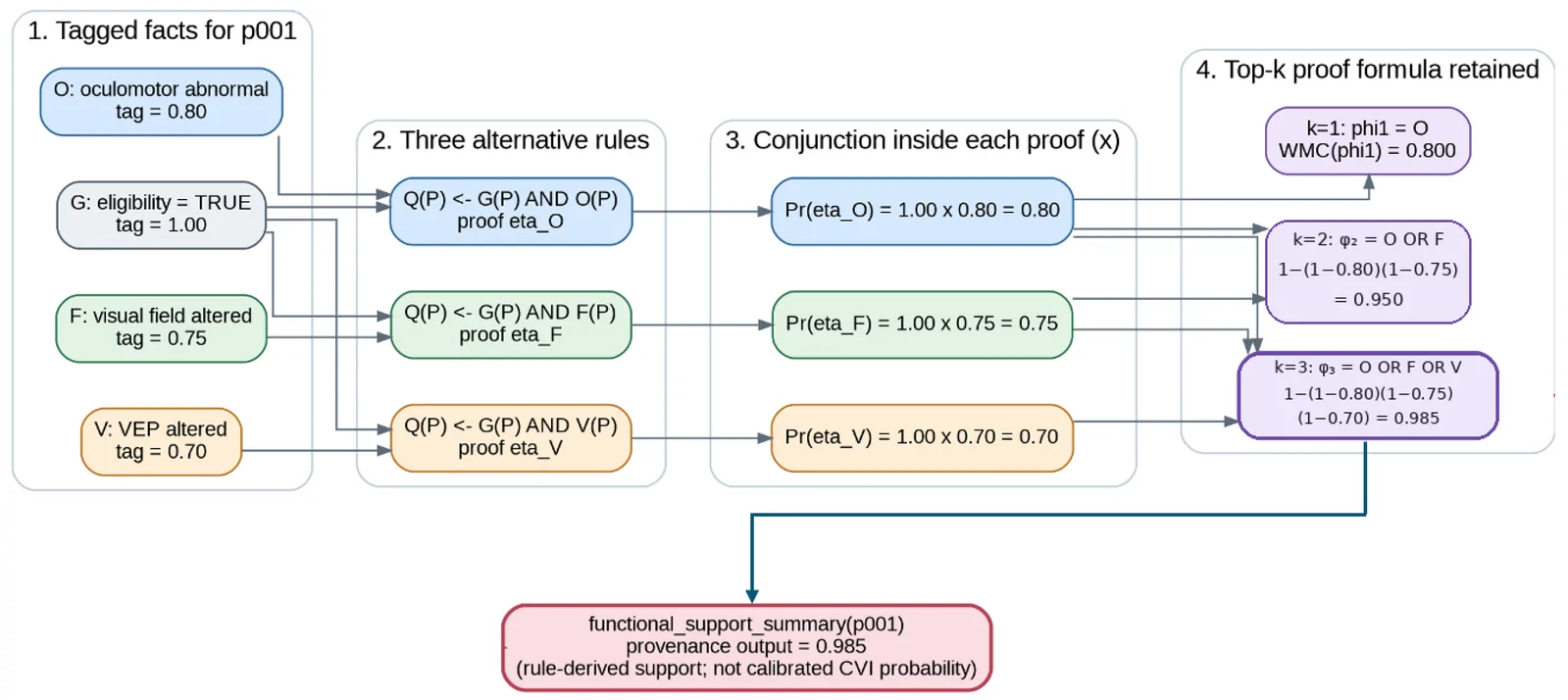
Worked patient-level provenance example. For a record satisfying the Boolean eligibility gate and the oculomotor (w=0.80), visual-field (w=0.75), and VEP (w=0.70) predicates, the three rules establish alternative proofs of the same functional-support relation. Top-*k* provenance retains one, two, or three already-satisfied derivations; weighted model counting of the retained proof formula yields support values of 0.800, 0.950, and 0.985, respectively. The figure exposes the transformation from tagged facts to rules, proof paths, and the final support value. These values are provenance-derived support under the specified tags and are not calibrated probabilities of CVI.

The system was designed as a transparent and interpretable decision-support framework rather than an autonomous diagnostic classifier. Its role was to transform heterogeneous clinical and instrumental findings into rule-supported profiles and to expose the facts and rules that supported each output. Because the reasoning is encoded through explicit clinically meaningful predicates rather than latent learned representations, an individual inference can be followed from observed facts through intermediate predicates and activated rules to alternative proof paths and the final queried relation. Provenance provides instance-level traceability of this derivation; it does not, by itself, establish clinical validity of the encoded knowledge. For the numerical top-*k* analysis reported here, the queried relation was functional_support_summary(P). It contains three alternative derivations, corresponding to oculomotor, visual-field, and VEP evidence after application of the non-weighted anterior_pathway_not_explanatory(P) eligibility gate. MRI-supported and higher-order profiles were queried separately and therefore do not alter the functional-only summary score. Figure 2 makes this derivational structure explicit. In a simple record, a checklist and the symbolic query may agree on the binary existence of at least one qualifying finding; however, the provenance representation additionally retains which rule paths support the relation, whether several paths converge, and which contextual relations are separately derivable. This distinction is the intended source of computational transparency and case-level inspectability rather than evidence of superior diagnostic accuracy.

### 2.8. Support Weights and Top-k Provenance

Positive anatomical, functional, and electrophysiological facts were assigned expert-defined support weights. These values were specified as proof-of-concept design parameters to demonstrate weighted provenance propagation; they were not learned from outcomes, calibrated as probabilities, or obtained through a formal expert-consensus procedure. Their purpose was to define an explicit and inspectable support hierarchy within the implemented rule system. Anterior pathway status was not treated as positive evidence and did not contribute numerically to the support score. Instead, the predicate anterior_pathway_not_explanatory(P) acted as a Boolean eligibility gate indicating that the available ocular, retinal, optic nerve, or chiasmal findings were not sufficient to explain the documented visual dysfunction. MRI abnormality was assigned anatomical support, whereas ocular motility, visual field, and VEP abnormalities were treated as functional or electrophysiological evidence domains. Table 4 lists the expert-defined weights and their roles.

The symbolic inference engine was executed using top-*k* proof provenance. For the functional CVI-compatible summary analysed in the present study, each satisfied evidence domain generated a proof with weight $w_{i}$. Available proof paths were ranked by weight and at most the first *k* paths were retained. Retained alternative proofs were aggregated using the probabilistic-OR provenance operation. This is a specified provenance aggregation over the supplied support tags; it does not assert statistical independence of the underlying clinical findings or establish clinical validity of the weights. The aggregation is defined as

(1) $$S_{k}=1-\prod_{i=1}^{\min(k,m)}\left(1-w_{i}\right),$$

where *m* is the number of available functional proof paths. Thus, for oculomotor (w=0.80), visual-field (w=0.75), and VEP (w=0.70) evidence, $S_{1}=0.800$, $S_{2}=1-\left(1-0.80\right)\left(1-0.75\right)=0.950$, and $S_{3}=1-\left(1-0.80\right)\left(1-0.75\right)\left(1-0.70\right)=0.985$. These are provenance-derived support values, not calibrated posterior probabilities. Increasing *k* does not add new patient evidence; it only allows additional already-satisfied proof paths to contribute to the summary. MRI support was represented in separate MRI-supported relations and was not included in the functional-only $S_{k}$ sequence reported below. Worked examples and the complete inference sequence are provided in Supplementary Sections S3 and S10, respectively.

### 2.9. Symbolic Rule Design

The rule base was designed in layers. The first layer derived domain profiles from observed facts:

(2) $$\begin{array}{cc} \mathrm{oculomotor\_profile}(P) & \leftarrow \mathrm{ocular\_motility\_abnormal\_observed}(P), \end{array}$$

(3) $$\begin{array}{cc} \mathrm{field\_profile}(P) & \leftarrow \mathrm{visual\_field\_altered\_observed}(P), \end{array}$$

(4) $$\begin{array}{cc} \mathrm{electrophysiology\_profile}(P) & \leftarrow \mathrm{vep\_altered\_observed}(P), \end{array}$$

(5) $$\begin{array}{cc} \mathrm{structural\_profile}(P) & \leftarrow \mathrm{mri\_abnormal}(P). \end{array}$$

The second layer defined functional evidence as the presence of at least one non-acuity functional domain:

(6) $$\begin{array}{cc} \mathrm{functional\_evidence}(P) & \leftarrow \mathrm{oculomotor\_profile}(P), \end{array}$$

(7) $$\begin{array}{cc} \mathrm{functional\_evidence}(P) & \leftarrow \mathrm{field\_profile}(P), \end{array}$$

(8) $$\begin{array}{cc} \mathrm{functional\_evidence}(P) & \leftarrow \mathrm{electrophysiology\_profile}(P). \end{array}$$

The main decision-support outputs were then defined as increasingly specific support profiles:

(9) $$\begin{array}{cc} \mathrm{cvi\_compatible\_profile}(P) & \leftarrow \mathrm{anterior\_pathway\_not\_explanatory}(P)\\ \; & \;\wedge \;\mathrm{functional\_evidence}(P), \end{array}$$

(10) $$\begin{array}{cc} \mathrm{mri\_supported\_profile}(P) & \leftarrow \mathrm{anterior\_pathway\_not\_explanatory}(P)\\ \; & \;\wedge \;\mathrm{structural\_profile}(P)\\ \; & \;\wedge \;\mathrm{functional\_evidence}(P), \end{array}$$

(11) $$\begin{array}{cc} \mathrm{two\_domain\_support}(P) & \leftarrow \mathrm{anterior\_pathway\_not\_explanatory}(P)\\ \; & \;\wedge \;\mathrm{oculomotor\_profile}(P)\\ \; & \;\wedge \;\mathrm{field\_profile}(P), \end{array}$$

(12) $$\begin{array}{cc} \mathrm{two\_domain\_support}(P) & \leftarrow \mathrm{anterior\_pathway\_not\_explanatory}(P)\\ \; & \;\wedge \;\mathrm{oculomotor\_profile}(P)\\ \; & \;\wedge \;\mathrm{electrophysiology\_profile}(P), \end{array}$$

(13) $$\begin{array}{cc} \mathrm{two\_domain\_support}(P) & \leftarrow \mathrm{anterior\_pathway\_not\_explanatory}(P)\\ \; & \;\wedge \;\mathrm{field\_profile}(P)\\ \; & \;\wedge \;\mathrm{electrophysiology\_profile}(P). \end{array}$$

MRI-enhanced multimodal support was defined when structural evidence and at least two functional domains were simultaneously present:

(14) $$\begin{array}{cc} \mathrm{mri\_plus\_two\_domain\_support}(P)\; & \leftarrow \mathrm{anterior\_pathway\_not\_explanatory}(P)\\ \; & \;\wedge \;\mathrm{structural\_profile}(P)\\ \; & \;\wedge \;\mathrm{oculomotor\_profile}(P)\wedge \;\mathrm{field\_profile}(P), \end{array}$$

(15) $$\begin{array}{cc} \mathrm{mri\_plus\_two\_domain\_support}(P)\; & \leftarrow \mathrm{anterior\_pathway\_not\_explanatory}(P)\\ \; & \;\wedge \;\mathrm{structural\_profile}(P)\\ \; & \;\wedge \;\mathrm{oculomotor\_profile}(P)\\ \; & \;\wedge \;\mathrm{electrophysiology\_profile}(P), \end{array}$$

(16) $$\begin{array}{cc} \mathrm{mri\_plus\_two\_domain\_support}(P)\; & \leftarrow \mathrm{anterior\_pathway\_not\_explanatory}(P)\\ \; & \;\wedge \;\mathrm{structural\_profile}(P)\\ \; & \;\wedge \;\mathrm{field\_profile}(P)\\ \; & \;\wedge \;\mathrm{electrophysiology\_profile}(P), \end{array}$$

(17) $$\begin{array}{cc} \mathrm{three\_domain\_mri\_support}(P)\; & \leftarrow \mathrm{anterior\_pathway\_not\_explanatory}(P)\\ \; & \;\wedge \;\mathrm{structural\_profile}(P)\\ \; & \;\wedge \;\mathrm{oculomotor\_profile}(P)\\ \; & \;\wedge \;\mathrm{field\_profile}(P)\\ \; & \;\wedge \;\mathrm{electrophysiology\_profile}(P). \end{array}$$

Discordance profiles were included because clinically relevant CVI-compatible findings may be missed by a single modality:

(18) $$\begin{array}{cc} \mathrm{mri\_function\_discordance}(P)\; & \leftarrow \mathrm{anterior\_pathway\_not\_explanatory}(P)\\ \; & \;\wedge \;\mathrm{mri\_normal}(P)\\ \; & \;\wedge \;\mathrm{functional\_evidence}(P), \end{array}$$

(19) $$\begin{array}{cc} \mathrm{vep\_function\_discordance}(P)\; & \leftarrow \mathrm{anterior\_pathway\_not\_explanatory}(P)\\ \; & \;\wedge \;\mathrm{vep\_normal\_observed}(P)\\ \; & \;\wedge \;\mathrm{oculomotor\_profile}(P), \end{array}$$

(20) $$\begin{array}{cc} \mathrm{vep\_function\_discordance}(P)\; & \leftarrow \mathrm{anterior\_pathway\_not\_explanatory}(P)\\ \; & \;\wedge \;\mathrm{vep\_normal\_observed}(P)\\ \; & \;\wedge \;\mathrm{field\_profile}(P). \end{array}$$

For the reported numerical top-*k* analysis, the functional summary was intentionally defined by three alternative derivations:

(21) $$\begin{array}{cc} \mathrm{functional\_support\_summary}(P) & \leftarrow \mathrm{anterior\_pathway\_not\_explanatory}(P)\\ \; & \;\wedge \;\mathrm{oculomotor\_profile}(P), \end{array}$$

(22) $$\begin{array}{cc} \mathrm{functional\_support\_summary}(P) & \leftarrow \mathrm{anterior\_pathway\_not\_explanatory}(P)\\ \; & \;\wedge \;\mathrm{field\_profile}(P), \end{array}$$

(23) $$\begin{array}{cc} \mathrm{functional\_support\_summary}(P) & \leftarrow \mathrm{anterior\_pathway\_not\_explanatory}(P)\\ \; & \;\wedge \;\mathrm{electrophysiology\_profile}(P). \end{array}$$

The MRI-supported, two-domain, MRI-plus-two-domain, three-domain-MRI, and discordance relations were retained as separate descriptive outputs. This separation makes explicit which relations contribute numerically to the functional top-*k* summary and which provide additional structural or contextual information.

### 2.10. Implementation and Reproducibility

The complete inference sequence is summarized in Supplementary Section S10. In brief, patient observations were encoded as positive, normal, or unavailable facts; the anterior-pathway eligibility gate was evaluated; domain profiles and support/discordance relations were derived; available proof paths were enumerated; the highest-weight paths were retained according to *k*; and retained alternatives were aggregated using the provenance operation defined above. The rule definitions, scoring equation, worked examples, evidence-domain combinations, and robustness analysis are reported in the Supplementary Materials. The implementation used the Scallop command-line interface (scli) with -p topkproofs and k=1,2,3. The frozen implementation is specified for the Scallop command-line interface (scli) version 0.1.4, matching the authors’ computational environment. The frozen rule implementation and executable command sequence are supplied with the Supplementary Materials. A final reproducibility check was completed using scli version 0.1.4: the released predicate-level facts reproduced the patient-level functional top-*k* outputs, the 54/62 CVI-compatible functional relations, the reported mean support values at k=1,2,3, and the higher-level support and discordance counts. The archived raw outputs and verification script are included in the Supplementary computational package (Supplementary Section S12).

## 3. Results

### 3.1. Cohort and Input Data

The symbolic inference system was applied to 62 pediatric patient records. Each record was encoded as a set of structured facts representing anterior pathway status, MRI status, ocular motility abnormalities, visual field alteration, and VEP alteration. For all 62 records, the available anterior pathway findings were classified as not sufficient to explain the documented visual dysfunction and therefore satisfied the non-weighted eligibility gate. MRI abnormalities were present in 47 patients (75.8%), whereas 15 patients (24.2%) had no MRI abnormality. Because no independent diagnostic reference standard was available, model derivation was not classified as a true-positive or false-positive result; the following analyses describe symbolic derivability and provenance behavior only.

### 3.2. Derived Evidence Domains

The first layer of inference derived domain-specific profiles from the input facts. The oculomotor profile was activated in 47 patients (75.8%), the visual field profile in 16 patients (25.8%), and the electrophysiology profile in 22 patients (35.5%). The summary output identified at least one CVI-compatible evidence path in 54 of 62 patients (87.1%). In this context, a CVI-compatible evidence path refers to satisfaction of the non-weighted anterior-pathway-not-explanatory gate together with at least one functional or instrumental abnormality, such as ocular motility abnormality, visual field alteration, or altered VEP. This output should be interpreted as rule-derived decision support rather than as an automated diagnosis. Table 5 summarizes the derived domain counts.

### 3.3. Evidence-Domain Combinations and Examination Availability

Because multimodal convergence depends on combinations rather than marginal domain counts alone, the joint distribution of the three functional evidence domains was examined. Oculomotor evidence alone occurred in 23/62 patients, visual-field evidence alone in 3/62, VEP evidence alone in 3/62, oculomotor plus visual-field evidence in 6/62, oculomotor plus VEP evidence in 12/62, visual-field plus VEP evidence in 1/62, and all three functional domains in 6/62. Eight patients had none of the three functional evidence paths. These combinations reproduce the marginal counts of 47 oculomotor, 16 visual-field, and 22 VEP profiles and the 54/62 overall functional derivability rate (Supplementary Table S2).

**Figure 3:**
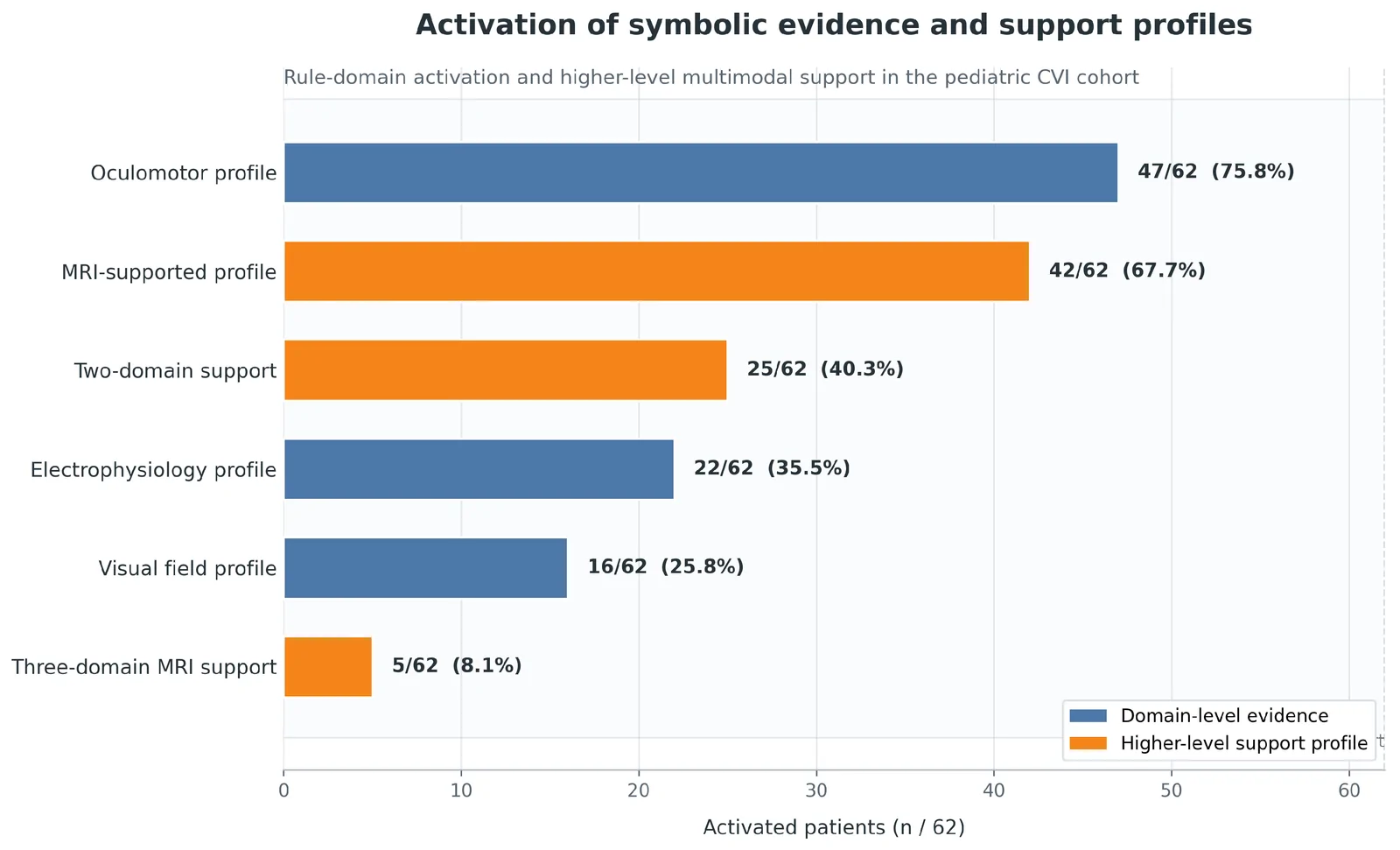
Derived symbolic evidence and support profiles in the pediatric CVI cohort. The figure shows the number and percentage of records satisfying each profile. Domain-level profiles represent oculomotor, visual field, and electrophysiological evidence. Higher-level support profiles represent MRI-supported evidence, two-domain functional support, and three-domain MRI-supported multimodal evidence.

Availability of the underlying examinations was heterogeneous. At least one visual-field result was recorded in 49/62 patients, including Goldmann perimetry in 9/62, the recorded clinical/monocular field assessment in 43/62, and Humphrey perimetry in 5/62. At least one VEP result was recorded in 50/62 patients; flash VEP was available in 44/62 and pattern VEP in 28/62. Missing entries were treated as unavailable evidence rather than as normal findings (Supplementary Table S3). Figure 3 summarizes the derived domain and higher-level support profiles.

### 3.4. Top-k Provenance Output

The inference engine was executed using top-k proof provenance with k=1, k=2, and k=3. With k=1, each patient retained the strongest single proof path. With k=2 and k=3, additional proof paths were retained when multiple evidence domains supported the same output.

With k=1, the summary relation was derived in 54 patients, with support scores ranging from 0.700 to 0.800 (Table 6). The most frequent score was 0.800, observed in 47 patients, corresponding to oculomotor evidence as the strongest retained path. Four patients had a score of 0.750, corresponding to visual field evidence as the strongest retained path. Three patients had a score of 0.700, corresponding to electrophysiological evidence as the strongest retained path.

When *k* was increased from 1 to 2, support scores increased in 25 of the 54 records with a derived functional path, indicating the presence of at least two retained functional evidence paths. When *k* was increased from 2 to 3, support scores increased further in 6 patients, indicating the presence of a third retained functional evidence path. The maximum support score increased from 0.800 with k=1 to 0.950 with k=2 and 0.985 with k=3. These values were recalculated after treating anterior pathway status as a non-weighted eligibility gate. The larger mean increase from k=1 to k=2 than from k=2 to k=3 reflects the observed combination structure: 25 records with a derived functional path contained at least two retained functional evidence paths, whereas only 6 contained all three and could gain a third path.

As an ablation of the expert-defined ranking, all three functional weights were also set to 0.75. Logical derivability was unchanged. Mean support changed from 0.791 to 0.750 for k=1, from 0.858 to 0.837 for k=2, and from 0.862 to 0.842 for k=3. For three retained functional paths, the score changed only from 0.985 to $1-{\left(1-0.75\right)}^{3}=0.984375$. Together with the ±10% perturbation analysis, this indicates that the multi-path convergence behavior is not dependent on the small differences among the expert-assigned functional weights; it does not constitute clinical validation of those weights.

### 3.5. Sensitivity of Support Scores to Expert-Defined Weights

Sensitivity of the provenance-derived support values to perturbation of the functional weights was examined in a dedicated robustness analysis. The complete procedure and numerical results are reported in Supplementary Section S8.

### 3.6. Patient-Level Provenance

Top-k provenance allowed patient-level outputs to be interpreted through their supporting rule paths. For example, patient p001 had a support score of 0.800 with k=1, 0.950 with k=2, and 0.985 with k=3. This increase reflects the retention of multiple supporting paths rather than a change in the underlying patient data. In practical terms, the system identified converging support from more than one evidence domain. Table 7 reports representative patient-level patterns.

### 3.7. Interpretation of Support Scores

The output CVI support scores are rule-derived values produced by the selected provenance model and are intended to quantify the degree of support assigned by the decision-support system to the CVI-compatible relation for an individual subject. Therefore, a score of 0.985 should not be interpreted as a 98.5% probability of CVI. Instead, it indicates strong support within the rule base due to multiple retained proof paths. The system should therefore be interpreted as a transparent and provenance-aware decision-support framework that organizes multimodal clinical evidence and exposes the rule paths supporting each output.

## 4. Discussion

### Original Contribution and Relation to Existing Approaches

The original contribution of the present work is not the invention of symbolic reasoning, provenance, or explainable artificial intelligence as general methods. Rather, it is the formalization of multimodal pediatric CVI reasoning as an explicit symbolic knowledge structure, together with patient-level provenance that retains alternative and convergent evidence paths and a reproducible computational feasibility evaluation of this representation. Scallop provides the underlying probabilistic deductive and provenance framework [25,26]; the contribution evaluated here is the CVI-specific clinical knowledge representation, rule architecture, and provenance-based decision-support layer built upon that framework.

The proposed system also differs in purpose from common comparator classes. A direct checklist can reproduce the binary existence of at least one qualifying functional finding but does not retain the derivational structure leading to the result. Conventional statistical or machine-learning classifiers generally estimate a learned mapping from input variables to a labelled target, whereas the present rules and weights were specified a priori and were not learned from the 62 study records. Post hoc explainable-AI methods seek to explain the behavior of an already fitted predictive model, whereas the present reasoning structure is explicit by construction. Hybrid or neurosymbolic systems may combine learned representations with symbolic inference; the current study deliberately evaluates only the symbolic reasoning layer, while learned sensor-level components remain prospective. Because no independent diagnostic reference standard was available, these methodological distinctions are not presented as evidence of superior predictive performance. Table 8 summarizes the architectural distinctions.

The intended clinical role is therefore not autonomous diagnosis. The system is intended to organize heterogeneous findings, expose which predefined evidence paths support a CVI-compatible relation, show when several paths converge, and retain missing or contextual/discordant information for clinician review. Whether this representation improves clinician interpretation, efficiency, diagnostic decisions, or patient outcomes has not been evaluated and requires prospective clinician-centered assessment.

CVI is a heterogeneous disorder in which visual dysfunction may involve acuity, visual field, fixation, ocular motility, visuospatial processing, and higher-order visual behavior. Current clinical approaches therefore emphasize multimodal assessment rather than reliance on a single diagnostic test [1,2,3,7,10]. Neuro-ophthalmological evaluation may include refraction, visual acuity testing, ocular motility assessment, visual field testing, OCT, MRI, and electrophysiology, but the availability and reliability of these examinations depend on age, cooperation, neurological status, and clinical indication [7,10,11]. The practical problem is that evidence is often heterogeneous, partially available, and distributed across several clinical and instrumental domains.

Clinical decision-support systems are designed to assist clinician reasoning by connecting patient-specific data with structured knowledge, while final interpretation remains a clinical responsibility [13,14]. This principle is particularly relevant in pediatric CVI, where assessment requires integration of ocular, neurological, functional, imaging, and electrophysiological evidence. At the same time, clinical AI systems require transparency, interpretability, validation, and workflow integration before they can be translated into routine practice [15,16,21,22]. Reporting frameworks for AI interventions and prediction models also emphasize clear description of intended use, data inputs, human interaction, and validation strategy [23,24,29]. These requirements are especially important when AI is used as decision support rather than as a fully autonomous system.

The aim of this study was to develop and test a symbolic AI inference system for decision support in the multimodal assessment of pediatric CVI. The system was designed to encode patient findings as structured facts, apply a non-weighted eligibility gate and expert-defined support weights, and return CVI-compatible evidence paths with top-k provenance. The goal was not to create an autonomous diagnostic classifier, but to provide a transparent, interpretable, and provenance-aware framework for organizing heterogeneous clinical and instrumental evidence.

The main finding was that the symbolic AI model identified at least one CVI-compatible evidence path in 54 of 62 patients (87.1%). In this context, a CVI-compatible evidence path refers to satisfaction of the non-weighted anterior-pathway-not-explanatory gate together with at least one functional or instrumental abnormality, such as ocular motility abnormality, visual field alteration, or altered VEP. This high derivability rate reflects the characteristics of the cohort, which included children already referred for neuro-ophthalmological evaluation and selected after exclusion of ocular bulb, retinal, optic nerve, and chiasmal pathology. Therefore, the result should not be interpreted as diagnostic performance. Rather, it shows that the symbolic inference system was able to identify rule-supported patterns of CVI-compatible evidence in the majority of records.

The strongest single proof path was usually the oculomotor evidence path after satisfaction of the anterior pathway eligibility gate. This is consistent with the high frequency of strabismus, fixation abnormalities, and ocular motility findings in the cohort, and with previous evidence that oculomotor abnormalities are common in children with CVI or brain-based visual dysfunction [7,10,30]. The visual field and electrophysiological domains were less frequently represented by derived proof paths, which likely reflects both true clinical heterogeneity and differences in test feasibility across pediatric patients. This point is important because the absence of a structured finding in retrospective data may reflect non-availability or limited reliability of a test, not necessarily normal function.

The binary 54/62 existence-of-path summary is equivalent to a direct count of records with at least one qualifying functional finding and is not presented as evidence of superiority over a checklist. The added computational contribution concerns the structure retained beyond that binary endpoint: explicit intermediate predicates, alternative and convergent derivations, and patient-level provenance. The top-k provenance analysis therefore provided the main computational contribution of the system. With k=1, each output retained only the strongest single proof path. Increasing *k* to 2 and 3 increased support scores in patients with converging evidence from multiple domains. This is potentially relevant to clinical review because CVI assessment rarely depends on one isolated measurement. Clinicians usually integrate functional vision, ocular motility, visual field, neuroimaging, and electrophysiological findings while considering the reliability of each test in a pediatric context [2,7,10]. Top-*k* provenance makes this computational distinction explicit by showing whether a support profile depends on one dominant evidence path or on several converging paths; whether this representation improves clinician interpretation or workflow was not evaluated in the present study. The completed reproducibility run also confirmed that increasing *k* does not itself increase every score: p001 increased from 0.800 to 0.950 to 0.985 because three functional derivations were available, whereas p002 remained at 0.800 for k=1,2,3 because only one functional derivation was established.

At the patient level, top-k provenance allowed the output to be interpreted through its supporting rule paths. For example, patient p001 had a support score of 0.800 with k=1, 0.950 with k=2, and 0.985 with k=3. This increase reflects the retention of multiple supporting paths rather than a change in the underlying patient data. In practical terms, the system identified convergent support from more than one evidence domain. Such output may be useful for clinical review because it helps distinguish between patients supported by a single dominant finding and patients supported by multiple distinct evidence domains.

The interpretation of support scores requires caution. The output CVI support scores are rule-derived values produced by the selected provenance model and are intended to quantify the degree of support assigned by the decision-support system to the CVI-compatible relation for an individual subject. They are not calibrated diagnostic probabilities. Therefore, a score of 0.985 should not be interpreted as a 98.5% probability of CVI. Instead, it indicates strong support within the rule base due to multiple retained proof paths. This distinction is essential for clinical use. The system should be interpreted as a decision-support framework that organizes evidence and exposes the rule paths supporting each output, not as a diagnostic device. This position is consistent with broader concerns in clinical AI: for high-stakes settings, models should be interpretable, clinically grounded, and validated for the way they will actually be used [21,22,31].

The proposed framework is also useful for identifying discordance. In CVI, normal or non-specific findings in one modality do not necessarily exclude clinically relevant visual dysfunction. For example, a child may show functional or oculomotor abnormalities despite non-altered MRI, or visual dysfunction despite non-altered VEP findings. Such discordance is not a failure of the reasoning process; it is a clinically relevant pattern that should prompt careful review. A symbolic inference system can make these patterns visible by separating multimodal support profiles from discordance profiles, thereby helping clinicians focus attention on cases where the evidence is incomplete or not fully concordant.

The use of a priori expert-defined support weights is an important methodological feature of this proof-of-concept implementation. The weights were not learned from labelled outcome data and were not intended to represent calibrated probabilities. They were assigned by Pensiero before analysis of the present cohort according to the relative clinical relevance of the evidence domains, while the rule structure was defined independently by Pensiero and Michieletto and subsequently formalised computationally. Anterior pathway status was separated from positive evidence and implemented as a non-weighted eligibility gate. MRI abnormality provided anatomical support, while ocular motility, visual field, and VEP abnormalities contributed functional or electrophysiological support. This separation prevents a constant exclusion criterion from artificially increasing every support score. The weighting scheme remains transparent and modifiable, but future versions should compare expert-defined values with weights calibrated against independent expert consensus, longitudinal outcomes, or external validation datasets.

The present system should therefore be considered a prototype symbolic inference layer intended for multimodal CVI decision support. The explicit rule structure provides computational transparency; the use of clinically meaningful predicates and explicit logical relations makes the reasoning computationally interpretable; and provenance provides traceability of the proof paths supporting an individual output. These properties do not by themselves establish clinical validity or clinical usefulness, and the effect of presenting such explanations to clinicians remains to be evaluated. Its current implementation uses structured clinical and instrumental variables as input facts. This design is intentionally modular and can be expanded. Quantitative MRI-derived lesion localization, OCT-derived retinal nerve fiber layer or ganglion cell measurements, eye-tracking features of fixation and pursuit, ocular-following responses, electroretinography, electrooculography, and VEP latency and amplitude could be encoded either as continuous probabilistic facts or as thresholded symbolic predicates, depending on their reliability and clinical meaning.

A further direction is neurosymbolic integration of images, signals, and structured data. In ophthalmology, image-derived outputs from OCT or other imaging modalities can be combined with symbolic reasoning to improve interpretability and clinical traceability [27]. In the spatially grounded framework of Inferrera et al., segmentation-derived anatomical priors guide OCT sign extraction, and continuous sign probabilities are propagated through expert-defined symbolic rules [28]. A similar strategy could be applied in pediatric CVI by allowing neural modules to generate candidate quantitative facts from OCT, MRI, eye-tracking, or electrophysiological recordings. Scallop supports neurosymbolic programs in which neural outputs participate in differentiable symbolic reasoning and rules can provide algorithmic constraints or supervision [25,26]. Such integration is prospective and was not evaluated in the present study. More broadly, recent clinical and methodological work has emphasized transparent reasoning chains, knowledge representation, multimodal integration, and explainability in healthcare AI [32,33,34].

A central limitation is the absence of an independent diagnostic reference standard. This is not simply a missing laboratory test: pediatric CVI is diagnosed through multidisciplinary clinical synthesis, and no single MRI, OCT, VEP, visual-field, or eye-movement measure constitutes a definitive gold standard. The present outputs therefore describe agreement with the encoded reasoning structure rather than agreement with a confirmed diagnosis. Diagnostic performance metrics such as sensitivity, specificity, predictive values, receiver-operating-characteristic curves, and calibration were not appropriate for this dataset. A subsequent prospective study should compare system outputs with diagnoses assigned independently by a multidisciplinary expert panel using predefined consensus criteria. The panel should be blinded to the symbolic support score during initial classification to avoid circular validation. External validation in an independent cohort and a clinician-facing usability/workflow evaluation are also required; neither was possible with the present retrospective dataset.

The present study used structured interpretations of clinical and instrumental examinations rather than raw sensor streams. Direct integration of quantitative video-oculography, OCT, MRI, or waveform-level electrophysiological features was not evaluated and should therefore be considered a future extension of the modular inference architecture rather than a contribution demonstrated by the present experiment.

Several next steps are necessary before clinical deployment. First, the rule base and support weights should be reviewed by a multidisciplinary expert panel and tested on additional cohorts. Second, the system should be evaluated prospectively to determine whether its outputs are useful for clinical workflow, multidisciplinary discussion, and follow-up planning. Third, the interface should present not only a final support score, but also the top proof paths, missing data, discordant domains, and suggested areas for clinical review. Fourth, future versions should compare structured symbolic input with quantitative sensor-derived and image-derived features to determine which data streams improve decision support. These steps are necessary because the present scores describe internal rule-derived support, not diagnostic accuracy, calibration, or clinical utility.

Overall, this study demonstrates that a symbolic inference system can organize heterogeneous pediatric CVI evidence into transparent, interpretable, and provenance-aware support profiles. The value of the approach lies not in replacing clinical judgment, but in making multimodal reasoning explicit. By exposing which evidence domains support an output, where multiple paths converge, and where discordance occurs, the system provides a foundation for AI-assisted decision support in pediatric CVI assessment.

## 5. Conclusions

This study demonstrates the feasibility and computational behavior of a prototype symbolic provenance framework for organizing multimodal evidence relevant to pediatric cerebral visual impairment. By combining structured clinical and instrumental variables with explicit rules, expert-defined support weights, and top-*k* proof provenance, the framework exposes which predefined evidence paths support each output and whether support arises from one or multiple domains. The reported support values are not calibrated diagnostic probabilities, and the present retrospective evaluation does not establish diagnostic accuracy or clinical utility, and it does not evaluate how clinicians use or perceive the presented explanations. Independent prospective validation against multidisciplinary consensus diagnosis, external-cohort evaluation, and clinician-facing assessment are required before claims of clinical decision-support performance can be made.

## Figures and Tables

**Table 1:** Clinical and etiological characteristics of the study cohort.

Characteristic	*n*/62	%
Male sex	39	62.9
Female sex	23	37.1
MRI abnormality	47	75.8
No MRI abnormality	15	24.2
Psychomotor delay	33	53.2
Epilepsy or seizures	25	40.3
Paresis or muscular hypotonia	22	35.5
Hearing impairment	7	11.3
Genetic etiology	34	54.8
Perinatal suffering/prematurity	13	21.0
Malformations	6	9.7
Meningoencephalitis	3	4.8
Other causes, mainly tumoral	6	9.7

**Table 2:** Structured variables considered for multimodal CVI reasoning.

Domain	Variables	Structured Representation
Demographics	Age, sex	Numeric and categorical facts
Etiology	Genetic, perinatal, malformation, meningoencephalitis, other	Binary etiological facts
Structural neuroimaging	Brain/orbital MRI; T1-weighted and T2-weighted anatomical images	MRI abnormality as present/absent
OCT imaging	OCT images	Retinal or optic nerve structural imaging facts when available
Anterior pathway not explanatory	Ocular bulb, retina, optic nerve, chiasm	Non-weighted eligibility predicate indicating that available anterior pathway findings were not sufficient to explain the visual dysfunction
Refraction	Astigmatism, hyperopia, myopia and severity	Refractive abnormality facts
Visual acuity	Monocular decimal acuity or Lea Gratings notation	Preserved or reduced acuity facts after refractive correction
Ocular motility	Exotropia, esotropia, vertical strabismus, nystagmus, fixation abnormality	Oculomotor/fixation facts
Visual field	Goldmann perimetry, attraction visual field (MonCvONE-PRO full-field projection perimeter; Metrovision, Pérenchies, France); Humphrey perimetry when feasible	Field status summarized as altered or not altered
Electrophysiology	Retimax flash VEP and pattern VEP	VEP alteration facts based on latency and amplitude

**Table 3:** Potential integration of sensor-derived features into the symbolic inference framework.

Sensor Modality	Potential Quantitative Features	Potential Role in the Inference System
Video-oculography	Fixation stability, saccade metrics, pursuit gain, ocular-following response amplitude and latency	Objective characterization of ocular motor control and cortical visual-motion processing; features may be encoded as continuous or probabilistic evidence facts.
Optical coherence tomography	Retinal layer thickness, ganglion cell complex, retinal nerve fiber layer, segmentation abnormalities, image-quality indices	Characterization of retinal and optic nerve structure and identification of findings that may explain, contribute to, or coexist with brain-based visual dysfunction.
Visual evoked potentials	Latency, amplitude, interocular asymmetry, waveform reproducibility, signal quality	Objective electrophysiological characterization of visual pathway function; waveform-derived features may complement or replace binary normal/altered coding.
Electroretinography and electrooculography	Retinal response amplitude and timing, retinal pigment epithelium function, recording-quality measures	Additional characterization of retinal function and support for differential interpretation of anterior and post-retinal contributions.
Magnetic resonance imaging	Lesion localization, regional volumes, white-matter integrity, visual pathway involvement	Quantitative anatomical support that may be combined with functional and electrophysiological evidence.

**Table 4:** Expert-defined support weights used in the initial symbolic inference implementation.

Input Fact	Weight	Role	Rationale
Anterior pathway not explanatory	Not weighted	Eligibility gate	Required before derivation of a CVI-compatible evidence path. This predicate does not provide positive evidence and does not contribute numerically to the support score.
MRI abnormality	0.85	Anatomical support	Provides structural evidence of brain involvement but does not alone define a functional CVI phenotype.
Ocular motility abnormality	0.80	Functional support	Captures strabismus, nystagmus, fixation, and ocular motility abnormalities frequently encountered in pediatric CVI assessment.
Visual field alteration	0.75	Functional support	Provides evidence of retrochiasmatic or functional visual pathway involvement, but availability and reliability depend on age and cooperation.
VEP alteration	0.70	Electrophysiological support	Provides objective visual pathway information, but normal VEP does not exclude CVI and abnormality may not fully explain functional vision.

**Table 5:** Derived evidence domains in the symbolic inference system.

Output Domain	*n*/62	%
Oculomotor profile	47	75.8
Visual field profile	16	25.8
Electrophysiology profile	22	35.5
CVI-compatible evidence path	54	87.1

**Table 6:** Top-k provenance summary for the CVI-compatible support output. The output was activated in 54 of 62 patients (87.1%) under all three top-k settings.

Provenance Setting	Score Range	Mean Support Score	Patients with Increased Score
k=1	0.700–0.800	0.791	–
k=2	0.700–0.950	0.858	25/54
k=3	0.700–0.985	0.862	6/54

**Table 7:** Representative patient-level top-k provenance patterns for the CVI-compatible support output.

Patient	k=1	k=2	k=3	Interpretation
p001	0.800	0.950	0.985	Three retained functional evidence paths: oculomotor, visual field, and electrophysiological.
p005	0.800	0.940	0.940	Two retained functional evidence paths: oculomotor and electrophysiological.
p012	0.750	0.925	0.925	Two retained functional evidence paths: visual field and electrophysiological.
p018	0.750	0.750	0.750	One retained visual field evidence path.
p033	0.700	0.700	0.700	One retained electrophysiological evidence path.
p062	0.800	0.950	0.950	Two retained functional evidence paths: oculomotor and visual field.

**Table 8:** Methodological comparison of the present symbolic framework with common decision-support approaches. The table describes architectural differences rather than comparative diagnostic performance.

Approach	Decision Function Learned from This Cohort?	Explicit Reasoning Structure	Alternative Derivations Retained	Patient-Level Provenance
Direct checklist	No	Limited to predefined items	No	No
Conventional statistical/ML classifier	Typically yes	Usually not as explicit rules	Not in the present sense	Model-dependent
Post hoc XAI + predictive model	Predictive model typically yes	Explanation follows prediction	Not in the present sense	Model-dependent explanation
Hybrid/neurosymbolic approach	May combine learned and symbolic components	Yes for symbolic component	Framework-dependent	Framework-dependent
Present symbolic CVI framework	No; rules fixed a priori	Yes	Yes	Yes

## Data Availability

The source pediatric clinical records and source clinical workbook are not publicly released. The Supplementary computational package does, however, include a pseudonymous record-level predicate encoding for all 62 records containing only the derived predicates required to execute the frozen symbolic analysis, together with the rule implementation, scoring/provenance specification, execution and verification scripts, aggregate tables, and archived inference outputs. The released predicate encoding is a derived computational representation and does not contain the source clinical records. Dissemination of these derived predicate-level inputs is covered by the applicable institutional authorization for this study. Access to the underlying clinical records remains subject to the applicable European and national data-protection framework, the study ethics approval and informed-consent conditions, and institutional governance.

## Supplementary Materials

The following supporting information can be downloaded at: https://www.mdpi.com/article/10.3390/s26216673/s1, Figure S1: Worked provenance calculation for p001; Table S1: Checklist comparison and targeted provenance ablations; Table S2: Joint combinations of the three functional evidence domains; Table S3: Availability of visual-field and VEP examinations in the retrospective source records; Table S4: Counts for layered and contextual symbolic relations.

## Abbreviations

The following abbreviations are used in this manuscript:

AI	Artificial intelligence
CDSS	Clinical decision-support system
CVI	Cerebral visual impairment
D	Diopter
MRI	Magnetic resonance imaging
OCT	Optical coherence tomography
RC	Ricerca Corrente
VEP	Visual evoked potential
VEPf	Flash visual evoked potential
VEPp	Pattern visual evoked potential
XAI	Explainable artificial intelligence
