# Supplementary material for: A Symbolic Inference System for AI Decision Support in Multimodal Assessment of Pediatric Cerebral Visual Impairment
Source: Sensors (Basel). 2026 Oct 22;26(21):6673. doi: 10.3390/s26216673 (PMC13765676; doi:10.3390/s26216673)
Supplement: Supplementary file 1 [file sensors-26-06673-s001.zip › CVI_Supplementary_Materials.pdf]

Supplementary Materials
A Symbolic Inference System for AI Decision Support in Multimodal Assessment of Pediatric Cerebral Visual Impairment

S1. Scope of the computational supplement

These Supplementary Materials provide the computational detail required to inspect and reproduce the revised symbolic experiment without releasing the source pediatric clinical records or source clinical workbook. The computational package includes a pseudonymous record-level predicate encoding for all 62 records containing only the derived predicates required by the frozen symbolic analysis. They specify the inference sequence, provenance aggregation, separation of functional and MRI-supported outputs, checklist/direct-count comparison, targeted provenance ablations, weight sensitivity, examination availability, evidence-domain combinations, illustrative test cases, and the frozen symbolic rule implementation.

The current study evaluates a symbolic reasoning layer operating on structured clinical observations. Direct processing of raw images, signals, or waveforms is prospective. The Scallop programming framework also supports neurosymbolic programs in which outputs of neural modules can participate in differentiable symbolic inference; this capability motivates later integration of raw or structured sensor data but is not evaluated in the present experiment.

S2. Inference, interpretability, and provenance

The model is not a data-driven classifier in which the decision is encoded primarily in latent learned parameters. Patient observations are represented as explicit facts, clinically meaningful intermediate predicates are derived by explicit rules, and one or more proof paths may lead to the same queried relation. Provenance retains the derivational structure of an individual inference. Thus, transparency refers to explicit access to facts, rules, weights, and aggregation; interpretability refers to reasoning expressed through clinically meaningful predicates and logical relations; and provenance provides instance-level traceability of the proof paths supporting an output. These computational properties do not establish clinical validity of the rules or weights.

S2.1 Provenance theory and its role in Scallop

In declarative logic programming, provenance records information about *how* a derived tuple or relation was obtained, rather than retaining only the Boolean fact that it was derivable. Scallop builds this idea on provenance semirings: relational tuples carry tags from a tag space T, and the tags are propagated alongside logical inference. The core algebra contains distinguished false and true tags (0 and 1), an addition-like operation $\oplus$ for alternative derivations, a multiplication-like operation $\otimes$ for conjunctive support within a derivation, and extensions for negation and fixed-point saturation. In this way, the same declarative rule structure can be executed with different provenance semantics for discrete, probabilistic, or differentiable reasoning [1, 2].

This distinction is central to the present application. A conventional Boolean execution answers whether a relation can be derived. Provenance can additionally retain information about the derivation(s) that made the relation true. For the present functional summary, oculomotor, visual-field, and VEP evidence constitute alternative proof paths to the same queried relation. The top-k proof setting limits the number of retained derivations and therefore controls reasoning granularity: $k = 1$ retains the strongest available proof, whereas larger k can preserve convergence of several already-satisfied paths. The patient data do not change when k changes.

The original Scallop work introduced provenance as a tunable framework that generalises probabilistic deductive reasoning while allowing scalable approximation of multiple proofs; the later language formulation generalised the same principle into a configurable provenance interface for discrete, probabilistic, and differentiable neurosymbolic programs [1, 2]. Importantly, a numeric provenance tag is not automatically a calibrated clinical probability. Its meaning is determined by the selected provenance semantics and the program in which it is used. Accordingly, the support values in this study are interpreted only as rule-derived support under the frozen aggregation specified below.

Alternative versus conjunctive derivations. Within a rule, conjunction requires all predicates in that proof to hold; across different rules for the same queried relation, the rules constitute alternative derivations. This makes provenance especially useful for multimodal clinical reasoning: one can distinguish a result supported by one domain from the same higher-level relation supported through several explicit routes. In the present implementation, the Boolean eligibility gate is required by each functional support rule, while O, F, and V provide the alternative weighted derivations. MRI-supported and contextual rules are deliberately queried as separate relations, preventing structural context from being mistaken for an additional term in the functional-only score.

Overlapping and subsumed proofs. A key reproducibility issue is whether a more complex derivation should numerically alter a simpler derivation that already proves the same relation. The revised analysis avoids ambiguity by freezing the functional summary to exactly three domain-level alternative rules. Layered MRI and multimodal conjunctions are exposed as separate named relations rather than additional proofs of `functional_support_summary(P)`. Consequently, they can enrich the explanation without silently inflating the functional score. This separation also makes the checklist ablation interpretable: the Boolean endpoint may remain identical while provenance retains derivational structure that the checklist discards.

Provenance, transparency, interpretability, and explanation. We use these terms distinctly. *Transparency* means that the facts, rules, weights, queries, and aggregation are explicitly inspectable. *Interpretability* means that intermediate predicates and logical relations have domain-level clinical meaning rather than being latent learned features. *Provenance* is the instance-level record of the derivational paths supporting a queried output. An *explanation* is a human-facing presentation of selected provenance information, for example the graph from observed facts to predicates, rules, proof paths, and the final relation. These computational properties can make reasoning auditable, but they do not establish that the rules, weights, or explanation are clinically valid or useful to clinicians; those questions require independent human-centred evaluation.

S2.2 From rules to proof formulas and weighted model counting

For reproducibility, it is useful to distinguish three objects that can otherwise be conflated: a *rule*, a *proof*, and the *provenance formula* of the queried tuple. Consider a query relation $Q(P)$ and three alternative rules

$$Q(P) \leftarrow G(P) \wedge O(P), \qquad Q(P) \leftarrow G(P) \wedge F(P), \qquad Q(P) \leftarrow G(P) \wedge V(P),$$

where G is the Boolean eligibility gate and O, F, V are tagged functional facts. Each satisfied rule produces one proof clause. In top-k proofs provenance, proof clauses are represented as conjunctions of the tagged input variables, while alternative rules are represented as a disjunction of proof clauses. Thus, before truncation,

$$\phi_Q = (G \wedge O) \vee (G \wedge F) \vee (G \wedge V).$$

For the present cohort G is a deterministic Boolean condition with tag 1 when satisfied. Therefore, for an eligible patient, multiplication by G leaves the probability/support of each proof unchanged and the formula reduces to

$$\phi_Q = O \vee F \vee V.$$

Scallop's top-k proof family retains at most k proof clauses ranked by proof probability. A proof probability is the product of the probabilities/tags of the literals in that conjunction. The retained Boolean formula is then evaluated by weighted model counting (WMC) under the provenance semantics [1, 2]. This distinction matters: WMC is performed on the Boolean formula over the underlying tagged facts, rather than treating already-overlapping proof clauses as if they were necessarily independent observations.

For a conjunction $\eta = v_1 \wedge \cdots \wedge v_r$ of positive tagged facts,

$$\Pr(\eta) = \prod_{j=1}^{r} \Pr(v_j).$$

For independent alternative base variables O, F, V, the WMC of $O \vee F \vee V$ is equivalently

$$1 - (1 - w_O)(1 - w_F)(1 - w_V),$$

which is the expression used for the functional example below. The same result can be written by inclusion–exclusion. Importantly, the numerical output is called a *provenance-derived support value* in this study. Although Scallop's probabilistic provenance performs a probability calculation over its tagged facts, the input weights used here were design weights rather than empirically calibrated disease probabilities; consequently the output must not be interpreted as the posterior probability that the child has CVI.

Why provenance is more than a graph. The provenance graph is only a visualisation of this algebra. The computational object is the proof formula and its tag propagation. The graph is useful because it exposes which base facts occur in each proof, which facts are shared across proofs, which alternative clauses survive the top-k truncation, and exactly which mathematical operations produce the returned value.

S2.3 Shared evidence and overlapping proofs

A second example illustrates why retaining the proof formula is important. Suppose a separate MRI-supported relation $R(P)$ has three rules $G \wedge M \wedge O$, $G \wedge M \wedge F$, and $G \wedge M \wedge V$, with $w_M = 0.85$. For p001 the individual proof products are

$$0.85 \times 0.80 = 0.680, \qquad 0.85 \times 0.75 = 0.6375, \qquad 0.85 \times 0.70 = 0.595.$$

However, these are not three independent pieces of structural evidence: every proof contains the same MRI variable M. The provenance formula is

$$(M \wedge O) \vee (M \wedge F) \vee (M \wedge V) = M \wedge (O \vee F \vee V).$$

Consequently, retaining all three clauses gives

$$\Pr(R) = 0.85\,[1 - (1-0.80)(1-0.75)(1-0.70)] = 0.85 \times 0.985 = 0.83725.$$

This is different from naively applying a probabilistic OR to the three proof products as if the shared MRI fact occurred independently three times. This example is included to clarify the role of provenance in preserving proof structure. In the revised study implementation, this MRI-supported relation is queried separately and does *not* alter the functional-only S_k values reported in the main manuscript.

S2.4 Relation to clinical reasoning structure

The graph is intended to illustrate the structure of the reasoning rather than claim that the program reproduces all aspects of human cognition. It formalises a common feature of clinical reasoning: the same higher-level conclusion may be approached through different findings or through convergence of several findings. The provenance therefore records not only whether a relation is derivable, but which explicit path or combination of paths supports that derivation.

S3. Frozen inference definition and score aggregation

The revised implementation separates the numerical functional top-k summary from MRI-supported higher-level profiles. The eligibility predicate `anterior_pathway_not_explanatory(P)` is Boolean and unweighted. Positive functional facts carry design weights 0.80 (oculomotor), 0.75 (visual field), and 0.70 (VEP). MRI abnormality carries weight 0.85 in MRI-supported relations but does not enter the functional-only top-k sequence reported in the main manuscript. Documented normal MRI and VEP findings are Boolean facts used in contextual/discordance relations. Missing or unrecorded examinations generate neither a positive nor a normal fact.

For a patient with m available functional proof paths and retained weights w_i, the reported functional support is

$$S_k = 1 - \prod_{i=1}^{\min(k,m)} (1 - w_i).$$

This is a provenance-derived support value under the specified aggregation and is not a calibrated probability of CVI. Increasing k does not create new clinical evidence; it permits additional already-satisfied alternative proofs to be retained.

S3.1 Worked provenance calculation: p001 from rules to output

Patient p001 provides a concrete end-to-end example. The eligibility gate is true, and all three functional observations are present. The relevant tagged facts are therefore

$$G = 1.00, \qquad O = 0.80, \qquad F = 0.75, \qquad V = 0.70.$$

The Scallop rules for the queried relation are

```
rel functional_support_summary(P) =
  anterior_pathway_not_explanatory(P) and oculomotor_profile(P)
rel functional_support_summary(P) =
  anterior_pathway_not_explanatory(P) and field_profile(P)
rel functional_support_summary(P) =
  anterior_pathway_not_explanatory(P) and electrophysiology_profile(P)
```

Step 1: construct the satisfied proofs. The three rules generate

$$\eta_O = G \wedge O, \qquad \eta_F = G \wedge F, \qquad \eta_V = G \wedge V.$$

Because conjunction corresponds to multiplication of positive literal weights within a proof,

$$\Pr(\eta_O) = 1.00 \times 0.80 = 0.80, \quad \Pr(\eta_F) = 1.00 \times 0.75 = 0.75, \quad \Pr(\eta_V) = 1.00 \times 0.70 = 0.70.$$

The proof ranking is therefore $\eta_O > \eta_F > \eta_V$.

Step 2: retain the top-k proof clauses. For $k = 1$, the retained formula is

$$\phi_1 = O,$$

and therefore $\mathrm{WMC}(\phi_1) = 0.800$.

For $k = 2$,

$$\phi_2 = O \vee F.$$

Its WMC is

$$\begin{aligned} S_2 &= \Pr(O \vee F) \\ &= \Pr(O) + \Pr(F) - \Pr(O \wedge F) \\ &= 0.80 + 0.75 - (0.80 \times 0.75) \\ &= 0.950. \end{aligned}$$

Equivalently, $S_2 = 1 - (1 - 0.80)(1 - 0.75) = 0.950$.

For $k = 3$,

$$\phi_3 = O \vee F \vee V.$$

The complete inclusion–exclusion calculation is

$$\begin{aligned} S_3 &= 0.80 + 0.75 + 0.70 \\ &\quad - (0.80 \times 0.75) - (0.80 \times 0.70) - (0.75 \times 0.70) \\ &\quad + (0.80 \times 0.75 \times 0.70) \\ &= 2.25 - 0.60 - 0.56 - 0.525 + 0.420 \\ &= 0.985. \end{aligned}$$

Equivalently,

$$S_3 = 1 - (1 - 0.80)(1 - 0.75)(1 - 0.70) = 0.985.$$

Step 3: recover the output tag. The query therefore returns p001 with a provenance-derived support of 0.800 at $k = 1$, 0.950 at $k = 2$, and 0.985 at $k = 3$. Figure S1 illustrates the calculation. The clinical observations have not changed across these executions. What changes is the maximum number of already-satisfied proof clauses retained in the provenance formula before WMC.

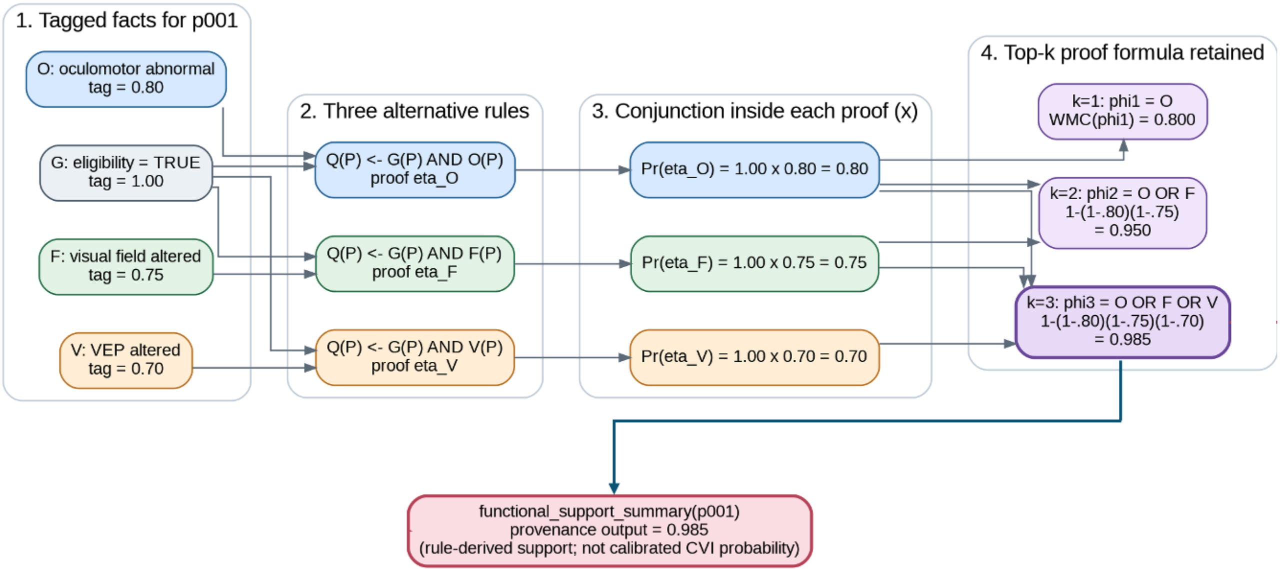

Figure S1: Worked provenance calculation for p001. Tagged facts satisfy three alternative rules. Conjunction inside each proof gives proof weights 0.80, 0.75, and 0.70. Top-k provenance retains one, two, or three proof clauses; WMC of the retained formula yields 0.800, 0.950, and 0.985. These values are provenance-derived support under the specified design weights, not calibrated probabilities of CVI.

ASCII representation of the same calculation.

```
p001 tagged facts
G = eligibility TRUE (1.00)
O = oculomotor abnormal (0.80)
F = visual field altered (0.75)
V = VEP altered (0.70)

RULE 1: Q <- G AND O    -> eta_O = G*O -> 1.00*0.80 = 0.80
RULE 2: Q <- G AND F    -> eta_F = G*F -> 1.00*0.75 = 0.75
RULE 3: Q <- G AND V    -> eta_V = G*V -> 1.00*0.70 = 0.70

Proof ranking: eta_O (0.80) > eta_F (0.75) > eta_V (0.70)

k=1: phi_1 = O
     WMC(phi_1) = 0.800

k=2: phi_2 = O OR F
     = 0.80 + 0.75 - (0.80*0.75)
     = 0.950

k=3: phi_3 = O OR F OR V
     = 0.80 + 0.75 + 0.70
       - (0.80*0.75) - (0.80*0.70) - (0.75*0.70)
       + (0.80*0.75*0.70)
     = 0.985

Returned support for Q(p001) at k=3: 0.985
NOT interpreted as Pr(CVI | patient data).
```

S3.2 Additional illustrative cases

The following examples are included to show why the system is not reduced to the binary question "is at least one abnormal finding present?".

Case	Functional paths	Structural/context	S_3	Interpretation of symbolic structure
p001	O+F+V	MRI abnormal	0.985	Three convergent functional derivations; separate MRI-supported relation also derivable.
p002	O only	MRI normal; VEP unavailable	0.800	One functional derivation; MRI-normal context does not negate the functional proof. Missing VEP creates no normal or abnormal VEP fact.
p004	O only	MRI abnormal; VEP documented normal	0.800	Functional proof remains derivable; documented normal VEP can participate in a separate contextual/discordance relation rather than cancelling O.
p007	O+F+V	MRI normal	0.985	Three convergent functional derivations despite normal MRI; a separate MRI/function discordance relation is derivable.
p013	none	MRI normal; VEP unavailable	–	No functional proof is established despite the eligibility gate.

These pseudonymous examples are provided for explanation of the computational behaviour. They are not released as a patient-level clinical dataset and are intentionally limited to the predicates required to illustrate inference.

S4. Checklist comparison and targeted provenance ablations

The binary summary "at least one CVI-compatible functional path" is equivalent, in this cohort, to a direct checklist count of records containing at least one qualifying functional finding after the constant eligibility gate. Both approaches identify 54/62 records. We therefore do not present 54/62 as evidence that the symbolic engine outperforms a checklist.

The added information appears when the same records are evaluated for reasoning structure rather than only binary existence of a path. Of the 54 records with at least one functional path, 29 have exactly one functional domain, 19 have exactly two, and 6 have all three. Consequently, a Boolean checklist collapses all 54 records to the same positive state, whereas provenance distinguishes one, two, and three alternative derivations and retains their identities.

Table S1: Checklist comparison and targeted provenance ablations.

Representation / ablation	Positive records	Convergence information	Information lost
Direct checklist (any O/F/V)	54/62	None	Path identity, convergence, provenance
$k = 1$ provenance	54/62	Strongest path only	Additional satisfied paths
$k = 2$ provenance	54/62	Up to two paths	Third path in 6 records
$k = 3$ provenance	54/62	Up to three paths	None among current three functional domains
Remove layered MRI outputs	54/62; functional summary unchanged	Functional provenance unchanged	Structural/contextual relations
Remove provenance; retain Boolean rules	54/62	None	Instance-level derivational trace

This ablation is deliberately aligned with the claimed contribution. It does not use a higher support score as evidence of improved diagnostic performance. Instead, it demonstrates what information is retained or lost when the layered rules and provenance mechanism are removed. MRI-supported and higher-order relations are separate outputs: they add structural/contextual descriptions but do not change the functional-only S_k values.

S5. Joint functional evidence-domain combinations

Table S2: Joint combinations of the three functional evidence domains.

Combination	n	%
Oculomotor only	23	37.1
Visual field only	3	4.8
VEP only	3	4.8
Oculomotor + visual field	6	9.7
Oculomotor + VEP	12	19.4
Visual field + VEP	1	1.6
All three functional domains	6	9.7
None of the three	8	12.9

The marginal counts are O=47, F=16, V=22, with at least one functional path in 54/62 records. Twenty-five records have at least two functional paths (6 OF + 12 OV + 1 FV + 6 OFV), whereas only six have all three. This explains why the cohort mean changes more from $k = 1$ to $k = 2$ than from $k = 2$ to $k = 3$.

S6. Examination availability and missing observations

Table S3: Availability of visual-field and VEP examinations in the retrospective source records.

Examination	n/62	%
Any visual-field information	49	79.0
Goldmann perimetry	9	14.5
Clinical/monocular field entry	43	69.4
Humphrey perimetry	5	8.1
Any VEP information	50	80.6
Flash VEP	44	71.0
Pattern VEP	28	45.2

The declarative representation distinguishes absence of a fact from a documented normal fact. If a test is unavailable or unrecorded, no positive or normal fact is generated for that domain; rules requiring that fact therefore cannot be established, while other derivations remain evaluable. A blank retrospective entry cannot reliably be subdivided into "not performed", "unavailable", "unreliable", or "not recorded", so those causes are not inferred retrospectively.

S7. Gate-negative, missing, normal, and contextual/discordant test cases

S7.1 Gate-negative synthetic test

```
Synthetic facts:
  anterior_pathway_not_explanatory = FALSE
  ocular_motility_abnormal_observed = TRUE
  visual_field_altered_observed = TRUE

Result:
  oculomotor_profile and field_profile can describe the observations,
  but functional_support_summary is NOT derivable because every
  support rule requires the eligibility predicate.
```

This synthetic case is included because the eligibility gate is constant in the retrospective cohort and therefore cannot be empirically exercised by the 62 records.

S7.2 Missing test

```
Eligibility = TRUE
Oculomotor abnormal = TRUE
VEP = unavailable/unrecorded

Derived: oculomotor proof
Not derived: VEP abnormal proof; VEP normal fact
Functional support remains evaluable through the available path.
```

S7.3 Apparently discordant modalities

```
Eligibility = TRUE
MRI normal = TRUE
```

```
Oculomotor abnormal = TRUE
Visual field altered = TRUE

Functional paths remain derivable.
MRI normal does not negate them.
A separate MRI/function contextual relation can also be derived.
```

The symbolic rules are not a hard decision tree in which one modality automatically cancels another. The program preserves the alternative derivations and can expose contextual relations for review.

S8. Weight and provenance robustness

The original functional weights (oculomotor 0.80, visual field 0.75, VEP 0.70) were assigned a priori as design parameters. The three functional weights were independently sampled from uniform intervals spanning ±10% of their original values in 10,000 simulations (random seed 42). Predicate activation is invariant to these positive perturbations because rule satisfaction depends on the presence of the encoded facts, not the magnitude of their positive support weights. For $k = 3$, the median Spearman correlation between perturbed and original patient scores was 1.000 (2.5th–97.5th percentile 0.818–1.000). The median mean absolute score change was 0.026 (2.5th–97.5th percentile 0.006–0.047). This analysis assesses numerical robustness only and does not validate the clinical appropriateness of the chosen weights.

Equal-weight ablation. To test whether the reported support behavior was primarily driven by the relative ordering of the expert-assigned functional weights, the provenance aggregation was repeated with identical weights of 0.75 for oculomotor, visual-field, and VEP evidence. This removes the expert-defined ordering while preserving approximately the scale of the original tags. Logical derivability and the number of available proof paths were unchanged. Across the 54 records with at least one functional path, mean support changed from 0.791 to 0.750 for $k = 1$, from 0.858 to 0.837 for $k = 2$, and from 0.862 to 0.842 for $k = 3$. For a record with all three functional paths, the original value of 0.985 became

$$1 - (1 - 0.75)^3 = 0.984375.$$

For two retained paths the equal-weight value was $1 - (1 - 0.75)^2 = 0.9375$, which lies between the original two-path values 0.925, 0.940, and 0.950. Thus, removing the relative ranking changed the numerical support values but preserved the principal effect of convergent derivations. This ablation does not validate the selected weights; it demonstrates that the multi-path convergence behavior is not dependent on the small differences among the three expert-assigned functional weights.

The provenance-setting ablation is reported in Section S4. Changing k from 1 to 2 affects 25/54 supported records because those records have at least two functional paths; changing k from 2 to 3 affects only the six records with all three paths. Thus, the effect of k is completely determined by the number of already-satisfied derivations and should not be interpreted as creation of additional evidence.

The probabilistic-OR aggregation is used here as a provenance support operation. It is not presented as a statistical model of causal independence among oculomotor, field, and VEP abnormalities. Correlated clinical domains may still constitute distinct symbolic derivations because the objective is to preserve the explicit reasoning paths; clinical calibration of their dependence requires an independent validation design.

S9. Counts for layered and contextual relations

Using the frozen revised predicates, the following descriptive relations are derivable in the 62 records:

Table S4: Counts for layered and contextual symbolic relations.

Relation	n/62
At least one functional path	54
MRI-supported functional profile	42
At least two functional domains	25
MRI + at least two functional domains	19
MRI + all three functional domains	5
MRI-normal with at least one functional path	12
Documented normal VEP with oculomotor and/or field path	22

These counts describe relations in the symbolic representation; they are not diagnostic performance measures and were not evaluated for clinician utility.

S10. Complete inference sequence

1. Encode each patient as structured facts. Positive abnormalities receive predefined support weights; documented normal findings required by contextual rules are Boolean; absent/unrecorded tests generate no fact.
2. Evaluate the Boolean anterior-pathway eligibility predicate. If false, relations requiring this predicate cannot be derived.
3. Derive oculomotor, visual-field, electrophysiological, and structural profiles.
4. Query `functional_support_summary(P)` for the three alternative functional proof paths.
5. Rank available functional proofs by support weight, retain at most k, and aggregate them using S_k.
6. Query MRI-supported, two-domain, MRI-plus-two-domain, three-domain-MRI, and contextual/discordance relations separately.
7. Return the derived relations, provenance paths, and support values for review. No diagnostic threshold is applied.

S11. Frozen Scallop rule implementation

The revised computational supplement uses the Scallop command-line interface `scli` with `-p topkproofs` and $k = 1, 2, 3$. The revised implementation was executed with the Scallop command-line interface `scli` version 0.1.4, using `-p topkproofs` and $k = 1, 2, 3$. The frozen rules defining the revised experiment are reproduced below. The accompanying computational files include the complete facts, a single-file program, and the command sequence used to query each relation.

```
rel oculomotor_profile(P) = ocular_motility_abnormal_observed(P)
rel field_profile(P) = visual_field_altered_observed(P)
rel electrophysiology_profile(P) = vep_altered_observed(P)
rel structural_profile(P) = mri_abnormal(P)

rel functional_evidence(P) = oculomotor_profile(P)
rel functional_evidence(P) = field_profile(P)
rel functional_evidence(P) = electrophysiology_profile(P)
```

```
rel functional_support_summary(P) =
  anterior_pathway_not_explanatory(P) and oculomotor_profile(P)
rel functional_support_summary(P) =
  anterior_pathway_not_explanatory(P) and field_profile(P)
rel functional_support_summary(P) =
  anterior_pathway_not_explanatory(P) and electrophysiology_profile(P)

rel cvi_compatible_profile(P) =
  anterior_pathway_not_explanatory(P) and functional_evidence(P)
rel mri_supported_profile(P) =
  anterior_pathway_not_explanatory(P) and structural_profile(P)
  and functional_evidence(P)

rel two_domain_support(P) =
  anterior_pathway_not_explanatory(P) and oculomotor_profile(P)
  and field_profile(P)
rel two_domain_support(P) =
  anterior_pathway_not_explanatory(P) and oculomotor_profile(P)
  and electrophysiology_profile(P)
rel two_domain_support(P) =
  anterior_pathway_not_explanatory(P) and field_profile(P)
  and electrophysiology_profile(P)

rel mri_plus_two_domain_support(P) =
  anterior_pathway_not_explanatory(P) and structural_profile(P)
  and oculomotor_profile(P) and field_profile(P)
rel mri_plus_two_domain_support(P) =
  anterior_pathway_not_explanatory(P) and structural_profile(P)
  and oculomotor_profile(P) and electrophysiology_profile(P)
rel mri_plus_two_domain_support(P) =
  anterior_pathway_not_explanatory(P) and structural_profile(P)
  and field_profile(P) and electrophysiology_profile(P)

rel three_domain_mri_support(P) =
  anterior_pathway_not_explanatory(P) and structural_profile(P)
  and oculomotor_profile(P) and field_profile(P)
  and electrophysiology_profile(P)

rel mri_function_discordance(P) =
  anterior_pathway_not_explanatory(P) and mri_normal(P)
  and functional_evidence(P)
rel vep_function_discordance(P) =
  anterior_pathway_not_explanatory(P) and vep_normal_observed(P)
  and oculomotor_profile(P)
rel vep_function_discordance(P) =
  anterior_pathway_not_explanatory(P) and vep_normal_observed(P)
  and field_profile(P)
```

S12. Reproducibility commands

The supplied run script queries the functional summary separately at $k = 1, 2, 3$ and the higher-level relations at $k = 3$:

```
for k in 1 2 3; do
  scli -p topkproofs -k "$k" -q functional_support_summary \
    cvi_inference_R1_single_file.scl
 done

for q in cvi_compatible_profile mri_supported_profile \
  two_domain_support mri_plus_two_domain_support \
  three_domain_mri_support mri_function_discordance \
  vep_function_discordance; do
  scli -p topkproofs -k 3 -q "$q" cvi_inference_R1_single_file.scl
 done
```

S12.1 Submitted computational reproducibility package

The Supplementary computational package contains the frozen Scallop rule definitions (`cvi_rules_R1.scl`), the pseudonymous predicate-level fact encoding used for the computational analysis (`cvi_facts_R1.scl`), a single-file executable implementation (`cvi_inference_R1_single_file.scl`), the Scallop execution script (`run_scallop.sh`), the output verification script (`verify_scallop_outputs.py`), the predicate-based robustness script (`sensitivity_from_predicates.py`), aggregate result tables, and the archived raw Scallop outputs. The final reproducibility run was completed with `scli` version 0.1.4. It reproduced 54/62 CVI-compatible functional relations; mean functional support values of 0.791, 0.858, and 0.862 at $k = 1, 2, 3$; 25 records with at least two functional domains; 19 with MRI plus at least two functional domains; 5 with MRI plus all three functional domains; 12 MRI/function discordance profiles; and 22 VEP/function discordance profiles. The worked p001 outputs were reproduced as 0.800, 0.950, and 0.985 at $k = 1, 2, 3$. In contrast, p002 remained at 0.800 for all three k settings because only one functional derivation was available. Thus, increasing k retains additional already-satisfied derivations when present; it does not mechanically increase every patient-level support value.

S13. Data governance and reproducibility without public clinical-data release

The source pediatric clinical records and source clinical workbook are not included in the public computational supplement. The released file `cvi_facts_R1.scl` contains a pseudonymous record-level predicate encoding for all 62 records, restricted to the derived predicates required to execute the frozen symbolic analysis. It does not contain the source clinical records. Dissemination of these derived predicate-level inputs is covered by the applicable institutional authorization for this study. Access to the underlying clinical records remains subject to the applicable European and national data-protection framework, the study ethics approval and informed-consent conditions, and institutional governance.

The released predicate facts are sufficient to reproduce the frozen Scallop inference, the patient-level top-k support outputs, the principal symbolic relation counts, and the weight-sensitivity/equal-weight analyses using `sensitivity_from_predicates.py`. Examination-availability counts and the derivation of the predicate encoding from the original clinical variables require the restricted source workbook and are therefore not independently regenerable from the public computational package. This distinction is intentional and is stated here to separate reproducibility of the released symbolic experiment from analyses that depend on the protected source clinical data.

Supplementary references

References

[1] Huang, J.; Li, Z.; Chen, B.; Samel, K.; Naik, M.; Song, L.; Si, X. Scallop: From Probabilistic Deductive Databases to Scalable Differentiable Reasoning. In *Advances in Neural Information Processing Systems*; **2021**; Volume 34, pp. 25134–25145. `https://papers.nips.cc/paper_files/paper/2021/hash/d367eef13f90793bd8121e2f675f0dc2-Abstract.html`.

[2] Li, Z.; Huang, J.; Naik, M. Scallop: A Language for Neurosymbolic Programming. *Proc. ACM Program. Lang.* **2023**, *7*, Article 166, 1463–1487. `https://doi.org/10.1145/3591280`.
